# Survival benefit and biomarker analysis of pyrotinib or pyrotinib plus capecitabine for patients with HER2-positive metastatic breast cancer: a pooled analysis of two phase I studies

**DOI:** 10.1186/s40364-023-00453-0

**Published:** 2023-02-20

**Authors:** Xiuwen Guan, Fei Ma, Qiao Li, Shanshan Chen, Bo Lan, Ying Fan, Jiayu Wang, Yang Luo, Ruigang Cai, Pin Zhang, Qing Li, Binghe Xu

**Affiliations:** Department of Medical Oncology and State Key Laboratory of Molecular Oncology, National Cancer Center / Cancer Hospital, Chinese Academy of Medical Sciences and Peking Union Medical College, No.17, PanjiayuanNanli, Chaoyang District, Beijing, 100021 China

**Keywords:** Metastatic breast cancer, HER2-positive, Pyrotinib, Survival, Concomitant mutations

## Abstract

**Background:**

Pyrotinib, a novel irreversible tyrosine kinase inhibitor (TKI), has demonstrated promising antitumor activity to improve the overall response rate and progression-free survival (PFS) in patients with HER2-positive metastatic breast cancer (MBC). However, the survival data of pyrotinib or pyrotinib plus capecitabine in HER2-positive MBC remains scarce. Thus, we summarized the updated individual patient data from the phase I trials of pyrotinib or pyrotinib plus capecitabine, to provide a cumulative assessment on long-term outcomes and associated biomarker analysis of irreversible TKIs in HER2-positive MBC patients.

**Methods:**

We performed a pooled analysis of the phase I trials for pyrotinib or pyrotinib plus capecitabine based on the updated survival data from individual patients. Next-generation sequencing was performed on circulating tumor DNA for predictive biomarkers.

**Results:**

A total of 66 patients were enrolled, including 38 patients from the phase Ib trial for pyrotinib and 28 patients from the phase Ic trial for pyrotinib plus capecitabine. The median follow-up duration was 84.2 months (95% CI: 74.7–93.7 months). The estimated median PFS in the entire cohort was 9.2 months (95% CI: 5.4–12.9 months) and median OS was 31.0 months (95% CI: 16.5–45.5 months). The median PFS was 8.2 months in the pyrotinib monotherapy cohort and 22.1 months in the pyrotinib plus capecitabine group, while the median OS was 27.1 months in the pyrotinib monotherapy group and 37.4 months in the pyrotinib plus capecitabine group. Biomarker analysis suggested that the patients harbored concomitant mutations from multiple pathways in HER2-related signaling network (HER2 bypass signaling pathways, PI3K/Akt/mTOR pathway and TP53) were observed with significantly poorer PFS and OS when compared to those with none or one genetic alteration (median PFS, 7.3 vs. 26.1 months, *P* = 0.003; median OS, 25.1 vs. 48.0 months, *P* = 0.013).

**Conclusions:**

The updated survival results based on individual patient data from the phase I trials of pyrotinib-based regimen revealed promising PFS and OS in HER2-positive MBC. Concomitant mutations from multiple pathways in HER2-related signaling network may be a potential efficacy and prognosis biomarker for pyrotinib in HER2-positive MBC.

**Trial registration:**

ClinicalTrials.gov. (NCT01937689, NCT02361112).

**Supplementary Information:**

The online version contains supplementary material available at 10.1186/s40364-023-00453-0.

## Introduction

The overexpression of human epidermal growth factor receptor 2 (HER2) or the amplification of HER2 gene accounts for 15–20% of breast cancer, which is associated with biologically aggressive disease and reduced overall survival (OS) before the introduction of HER2-targeted therapies [1]. The advent of trastuzumab and other HER2-targeted therapies have changed the standard-of-care for patients with HER2- positive breast cancer and significantly improved the survival outcome. The standard first-line therapy for HER2-positive metastatic breast cancer is the combination of anti-HER2 monoclonal antibody trastuzumab and pertuzumab plus chemotherapy based on the results of CLEOPATRA study [2–4]. After first-line trastuzumab-based therapy, HER2 antibody-drug conjugate trastuzumab deruxtecan was the recommended standard second-line therapy [2, 4]. In the third line or beyond therapy for HER2-positive metastatic breast cancer, small-molecule tyrosine kinase inhibitors (TKIs) that target HER2 and other HER family receptors play an important role in this setting [2, 4, 5].

The small molecule TKIs diffuse across the cell membrane and bind to the cytoplasmic catalytic kinase domain of the HER family proteins and inhibit the interaction with adenosine triphosphate (ATP), thus blocking tyrosine phosphorylation and activation of downstream signaling cascades, and leading to a decreased growth and proliferation of the cancer cells [5, 6]. Pyrotinib is a novel, oral, irreversible pan-ErbB TKI that potently inhibits epidermal growth factor receptor (EGFR)/HER1, HER2, and HER4. In the phase I to phase III studies [7–10], pyrotinib was demonstrated promising antitumor activity in patients with HER2-positive metastatic breast cancer, which was approved by the National Medical Products Administration in China for treatment in combination with capecitabine in patients with relapsed or metastatic HER2-positive breast cancer previously treated with anthracycline or taxane. Pyrotinib was initially evaluated as a monotherapy in a single-arm, phase I dose escalation study in HER2-positive metastatic breast cancer, with an overall response rate (ORR) of 50.0% and a median progression-free survival (PFS) of 35.4 weeks (95% CI, 23.3 to 40.0 weeks) [7]. Diarrhea was the only grade 3 pyrotinib-related AE (13.2% [five of 38]) in this phase I trial and was managed by appropriate medication (such as loperamide). Another phase I study to assess the safety, tolerability, pharmacokinetics, and antitumor activity of pyrotinib combined with capecitabine observed an ORR of 78.6% and a median PFS of 22.1 months (95% CI: 9.0 to 26.2 months) in patients with HER2-positive metastatic breast cancer [8]. In this study, grade 3 treatment-related adverse events (AE) occurred in 12 patients (42.9%), and anemia (14.3%) and diarrhea (10.7%) were the most common grade 3 AEs.

For metastatic breast cancer, the primary therapeutic goals focus on the prolongation of survival and the maintenance of quality of life (QoL) [2, 4]. However, current data on the survival outcome of HER2-positive metastatic breast cancer patients receiving irreversible TKIs or TKIs combined with chemotherapy was still limited. In the phase III NALA Trial for HER2-positive metastatic breast cancer [11], irreversible pan-ErbB TKI neratinib plus capecitabine demonstrated significant improvement in the PFS, but no significant benefit gained in OS compared to lapatinib plus capecitabine (24.0 months vs 22.2 months, *P* = 0.2086) [11]. While in the phase III PHOEBE study [10], pyrotinib plus capecitabine achieved clinically and statistically significant improvement of PFS and a trend of OS benefits based on the interim analysis data (with the cutoff of March 31, 2019), when compared with lapatinib plus capecitabine (NR vs 26.9 months, HR = 0.69, 95% CI:0.48–0.98, *P* = 0.02). Thus, the survival benefit of TKIs combined with chemotherapy was uncertain in breast cancer patients. Meanwhile, the survival data of pyrotinib or pyrotinib plus capecitabine in HER2-positive metastatic breast cancer is still lacking.

Therefore, we conducted a pooled analysis of the updated individual patient data from the phase I trials for pyrotinib monotherapy and pyrotinib plus capecitabine in HER2-positive metastatic breast cancer, to provide a cumulative assessment of updated long-term outcomes for irreversible TKI. Additionally, biomarker analysis was performed to identify potential predictors of efficacy and prognosis for pyrotinib-based regimens.

## Materials and methods

### Study design

This study was a pooled analysis of individual patient data from the phase Ib trial for pyrotinib [7] (NCT01937689) and the phase Ic trial for pyrotinib plus capecitabine [8] (NCT02361112), that both enrolled patients in the National Cancer Center of China. Original patient-level data was collected including individual clinicopathological characteristics, treatment, clinical outcomes and updated survival outcomes.

Both the two trials were under the traditional 3 + 3 design for dose escalation. In the phase Ib trial for pyrotinib [7], patients with HER2-positive metastatic breast cancer received pyrotinib monotherapy (80 mg, 160 mg, 240 mg, 320 mg, 400 mg and 480 mg) orally once per day, while in the phase Ic trial for pyrotinib plus capecitabine [8], patients underwent pyrotinib (160 mg, 240 mg, 320 mg, and 400 mg) orally once per day for 21-day cycles in combination with capecitabine (1000 mg/m^2^ orally twice per day on days 1 to 14).

The protocol was approved by the Institutional Review Board of National Cancer Center / Cancer Hospital, Chinese Academy of Medical Sciences and Peking Union Medical College. This study was conducted in accordance with the International Conference on Harmonization Guideline for Good Clinical Practice and the ethical principles in the Declaration of Helsinki. Each of the patients provided written informed consent before participation. The study protocol was approved by all participating investigators.

### Patient eligibility

Patients were eligible for enrollment if they (1) had a pathologically confirmed diagnosis of HER2-positive (defined as either an immunohistochemistry score of 3+ or 2+ together with HER2 gene amplification by fluorescence in situ hybridization) relapsed or metastatic breast cancer, (2) aged between 18 and 70 years, (3) had a Eastern Cooperative Oncology Group (ECOG) performance status of 0 or 1, (4) had at least one measurable lesion defined by revised Response Evaluation Criteria in Solid Tumors guidelines version 1.1 (RECIST v1.1), and (5) had adequate bone marrow and organ function. Patients were excluded if they had (1) any anticancer treatment given within 4 weeks before enrollment, or (2) previous treatment with anti-HER2 TKIs, or (3) previous ineffective standard capecitabine treatment (ie, disease progression during capecitabine treatment or a response lasting less than 3 months after capecitabine treatment discontinuation), or (4) previous effective standard capecitabine treatment less than 6 months before enrollment, or (5) history of brain metastasis.

### Endpoints

The survival endpoints in this analysis were OS and PFS. OS was defined as the time from the date of enrollment to the date of death due to any cause. PFS was defined as the time from the date of enrollment to the date of first documentation of disease progression confirmed by blinded independent central review or date of death due to any cause, whichever occurred first.

### Biomarker analysis

Next-generation sequencing of targeted gene sequencing panel (1021 genes) was performed on circulating tumor DNA (ctDNA) and genomic DNA of baseline peripheral blood samples for predictive biomarkers in the phase I trials for pyrotinib and pyrotinib plus capecitabine. All biomarker analyses were prospectively planned, and informed consents for blood collection were obtained from patients. Detailed protocols for ctDNA sequencing are provided in the supplemental materials [7, 8].

### Statistical analysis

All the patients recruited in these two phase I trials were considered assessable and were included in this pooled dataset. The clinicopathological characteristics of the recruited patients were described in percentages of categorical variables. *χ*2 test or Fisher’s test were used to compare the distribution of clinicopathological characteristics between different groups. Kaplan-Meier estimations were utilized to report outcomes of PFS and OS, and a stratified Cox proportional hazards model was used to estimate hazard ratios (HR) and 95% confidence intervals (CI). All statistical tests were two-sided, and *P* values below 0.05 were considered statistically significant. All analyses were performed with SPSS software (version 23.0, SPSS Inc., Chicago, IL, USA) and R software (version 4.0.5, Institute for Statistics and Mathematics, Vienna, Austria). Kaplan-Meier survival plots were performed, and the number at risk was determined using MedCalc (version 20.022, MedCalc Software Bvba, Ostend, Flanders).

## Results

### Patient characteristics

**Fig. 1:**
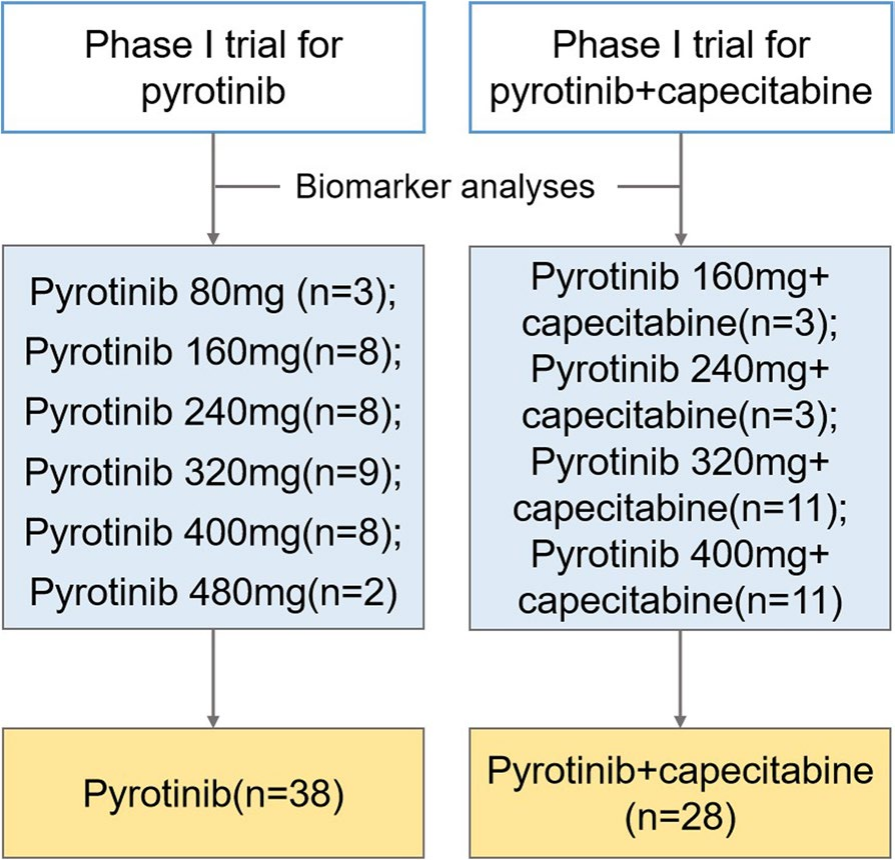
Study flowchart

A total of 66 patients were enrolled in the analyzed including 38 patients from the phase Ib trial for pyrotinib and 28 patients from the phase Ic trial for pyrotinib plus capecitabine (Fig. 1). The baseline demographic and clinical characteristics of enrolled patients are presented in Table 1. The median age at the time of enrolment was 47.5 years (range: 24 to 67). In the analysis, nearly half of the participants (34, 51.5%) were hormone receptor (HR)-positive patients and almost 80% of the patients (51 patients) had visceral metastases. Among them, 89.4% (59 patients) of them had received an anthracycline, and 98.5% (65 patients) had received a taxane. Besides that, 65.2% of the patients (43 patients) had received prior trastuzumab treatment, and more than half of them (27 patients, 62.8%) have had trastuzumab for metastatic disease. Among the 16 patients who received trastuzumab only in adjuvant/neoadjuvant setting, 11 patients were resistant to previous trastuzumab (defined as relapsed during adjuvant treatment or within 6 months after adjuvant trastuzumab). No significant difference was observed between patients from phase I trial for pyrotinib and those from phase I trial for pyrotinib plus capecitabine in the demographic characteristics regarding age, menstrual status, ER status, PR status, position of metastatic site, previous chemotherapy and prior trastuzumab treatment.

**Table 1:** Baseline demographic and clinical characteristics of enrolled patients in the analysis

	Pyrotinib monotherapy group (*n* = 38)	Pyrotinib plus capecitabine group (*n* = 36)	*χ*^*2*^	*P* value
Median age, years (range)			0.122	0.727
≤ 45	16(42.1)	13(46.4)		
>45	22(57.9)	15(53.6)		
Menstrual status			0.041	0.840
Premenopausal	24(63.2)	17(60.7)		
Postmenopausal	14(36.8)	11(39.3)		
ECOG			2.507	0.113
0	35(92.1)	22(78.6)		
1	3(7.9)	6(21.4)		
ER status			1.861	0.173
Positive	20(52.6)	10(35.7)		
Negative	18(47.4)	18(64.3)		
PR status			3.821	0.051
Positive	20(52.6)	8(28.6)		
Negative	18(47.4)	20(71.4)		
Tumor site			0.143	0.705
Visceral	30(78.9)	21(75.0)		
Nonvisceral	8(21.1)	7(25.0)		
Prior taxane treatment, n (%)	38(100)	27(96.4)	1.378	0.240
Prior anthracycline treatment, n (%)	35(92.1)	24(85.7)	0.694	0.405
Prior trastuzumab treatment, n (%)			0.482	0.923
Trastuzumab-pretreated for metastatic disease only	12(31.6)	8(28.6)		
Trastuzumab-pretreated in the adjuvant/neoadjuvant setting only	10(26.3)	6(21.4)		
Trastuzumab-pretreated in both adjuvant/neoadjuvant setting and metastatic setting	4(10.5)	3(10.7)		
Non	12(31.6)	11(39.3)		

### Survival outcomes for pyrotinib-based regimen

**Fig. 2:**
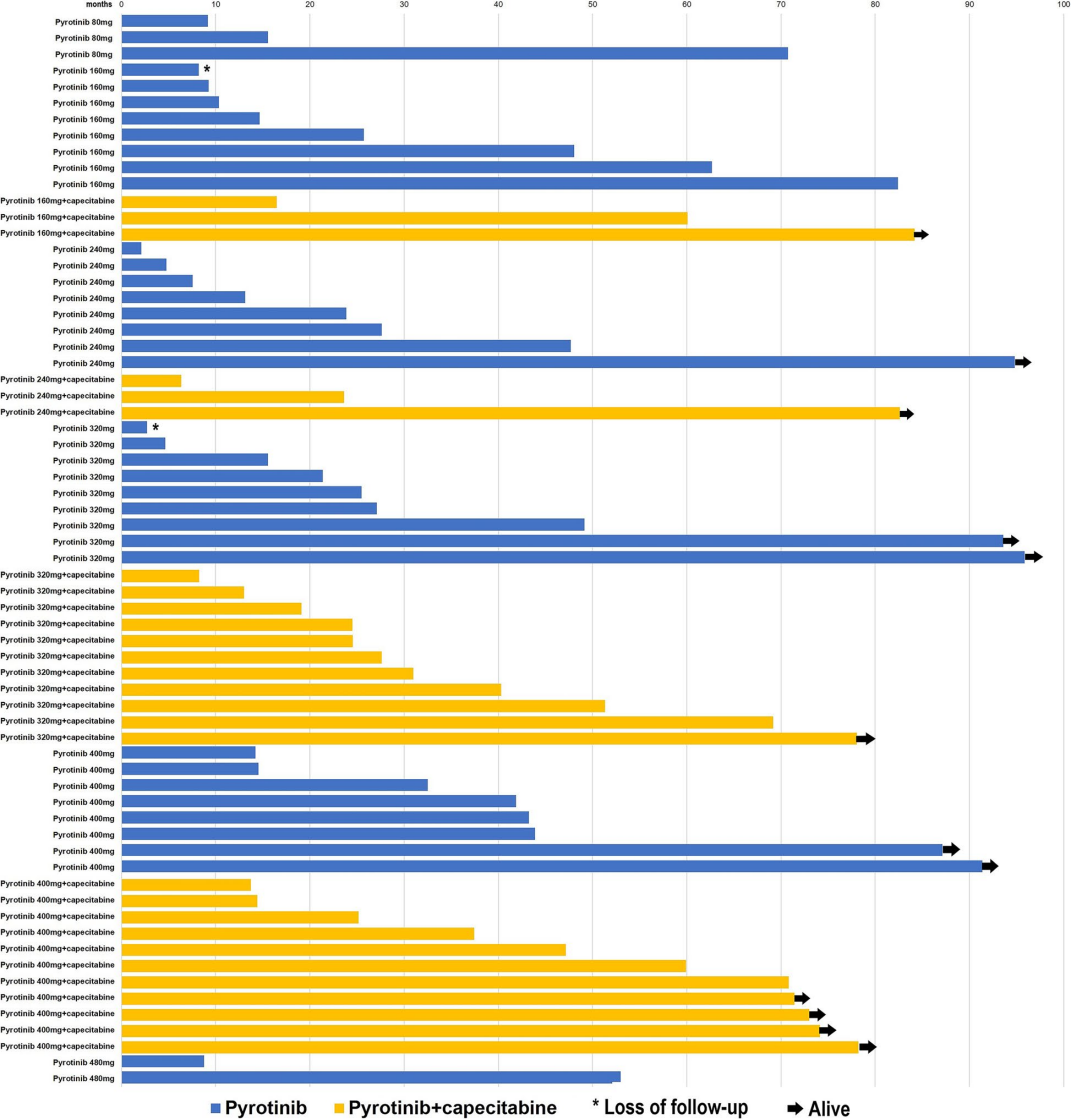
Overall survival duration of patients in different dose cohorts for patients receiving pyrotinib, or pyrotinib plus capecitabine

The median follow-up duration for OS was 84.2 months (95% CI: 74.7–93.7 months) and for PFS was 73.0 months (95% CI: 69.8–76.2 months). Death was reported in 52 participants, including 31 patients in the pyrotinib monotherapy cohort and 21 patients in pyrotinib plus capecitabine cohort (Fig. 2). At the time of data cutoff (October, 2021), four patients were still ongoing the treatment in the trial cohorts including one from the phase Ib trial for pyrotinib, and three from the phase Ic trial for pyrotinib plus capecitabine.

The estimated median PFS in the entire cohort was 9.2 months (95% CI: 5.4–12.9 months) and the median OS was 31.0 months (95% CI: 16.5–45.5 months). The median PFS was 8.2 months (95% CI: 5.6–10.9 months) in the pyrotinib monotherapy cohort and 22.1 months (95% CI: 14.3–29.9 months) in the pyrotinib plus capecitabine cohort, while the median OS was 27.1 months (95% CI: 21.6–32.5 months) in the pyrotinib monotherapy cohort and 37.4 months (95% CI: 12.2–62.7 months) in the pyrotinib plus capecitabine cohort (Table 2). The survival outcome of different doses of pyrotinib or pyrotinib plus capecitabine in the phase I trials was described in Table 2. For the patients in the pyrotinib 400 mg dose cohort, which was recommended for the phase II and phase III trials, the median PFS was 13.7 months (95% CI: 7.3–20.2 months) in the pyrotinib monotherapy group (*n* = 8) and 29.9 months (95% CI: 21.2–38.6 months) in the pyrotinib plus capecitabine group (*n* = 11). Regarding OS outcome for pyrotinib 400 mg dose cohort, the median OS was 41.9 months (95% CI: 27.0–56.8 months) in the pyrotinib monotherapy group (*n* = 8) and 59.9 months (95% CI: 23.9–95.9 months) in the pyrotinib plus capecitabine group (*n* = 11). The survival duration of patients in different dose cohorts for patients receiving pyrotinib monotherapy and pyrotinib plus capecitabine was depicted in Fig. 2.

**Table 2:** The survival outcome of different doses of pyrotinib or pyrotinib plus capecitabine in the phase I trials

	*n*	median PFS (months)	median OS (months)
The entire cohort	66	9.2(5.4–12.9)	31.0(16.5–45.5)
Pyrotinib	38	8.2(5.6–10.9)	27.1(21.6–32.5)
Pyrotinib 80 mg	3	5.5	15.5
Pyrotinib 160 mg	8	7.3	25.7
Pyrotinib 240 mg	8	3.3	13.1
Pyrotinib 320 mg	9	7.3	25.5
Pyrotinib 400 mg	8	13.7	41.9
Pyrotinib 480 mg	2	1.4	8.8
Pyrotinib +capecitabine	28	22.1(14.3–29.9)	37.4(12.2–62.7)
Pyrotinib 160 mg + capecitabine	3	26.1	60.1
Pyrotinib 240 mg + capecitabine	3	6.9	23.6
Pyrotinib 320 mg + capecitabine	11	16.4	27.6
Pyrotinib 400 mg + capecitabine	11	29.9	59.9

### Biomarker analysis

**Fig. 3:**
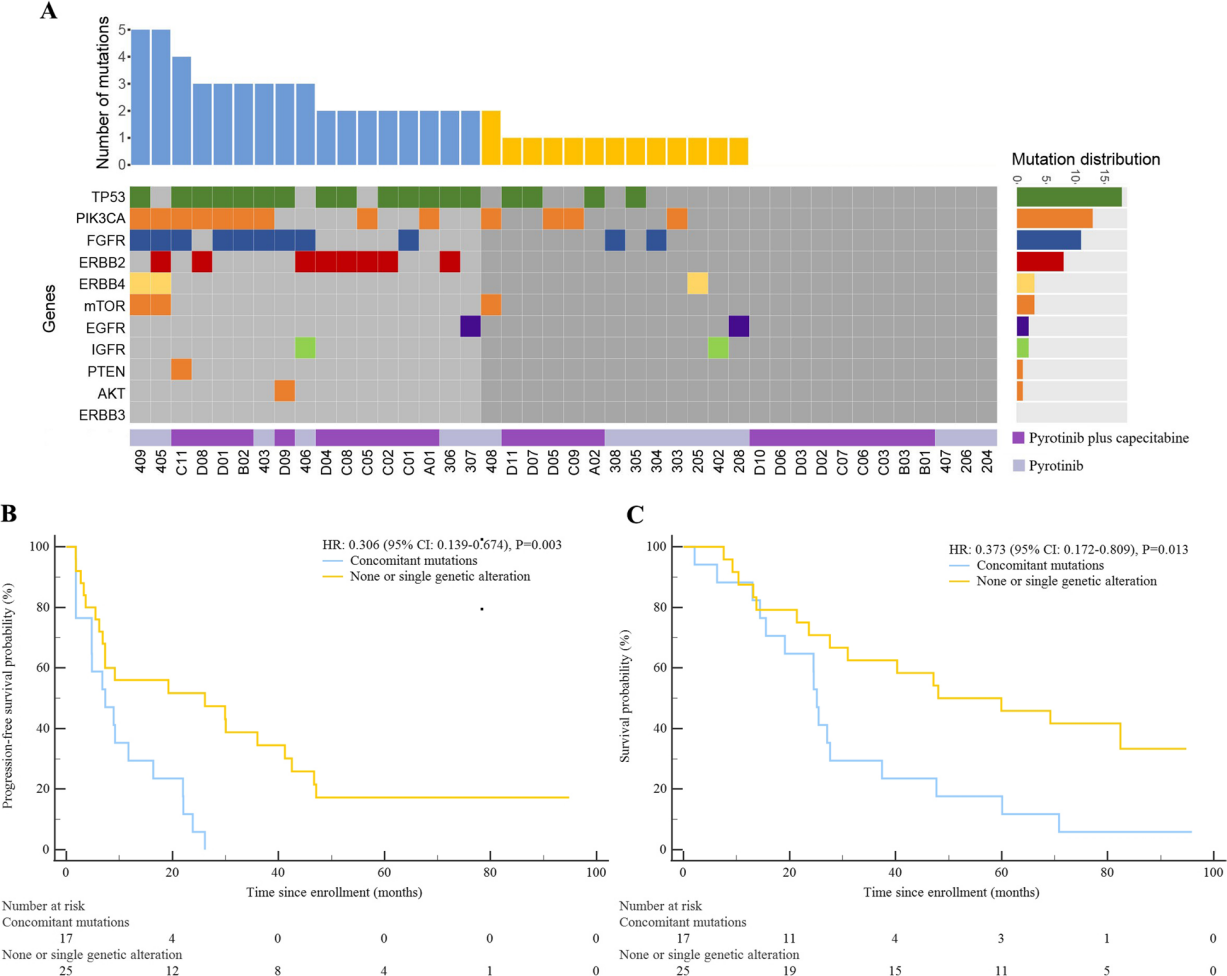
Mutation distribution of HER2 bypass signaling pathway, PI3K/Akt/mTOR pathway and TP53 in ctDNA of baseline samples and its association with survival outcome. **A** Mutation distribution of HER2 bypass signaling pathway, PI3K/Akt/mTOR pathway and TP53 in ctDNA of baseline samples. **B** Kaplan-Meier estimates of progression-free survival comparing patients harbored concomitant mutations from multiple pathways in above HER2-related signaling network to those with none or one genetic alteration. **C** Kaplan-Meier estimates of overall survival comparing patients harbored concomitant mutations from multiple pathways in above HER2-related signaling network to those with none or one genetic alteration

All genetic alterations of HER2-related signaling network, containing HER2 bypass signaling pathways (including EGFR, FGFR, IGFR, ERBB2, ERBB3, ERBB4), PI3K/Akt/mTOR pathway (PIK3CA, AKT, mTOR, PTEN) and TP53 were analyzed for baseline blood samples of 42 patients in the phase I trials for pyrotinib and pyrotinib plus capecitabine (Fig. 3A). In ctDNA mutational analysis, the commonly mutated genes were TP53 (42.9%, 18/42), PIK3CA (31.0%,13/42) and FGFR (26.2%,11/42).

On basis of updated survival data in the whole cohort, the patients harbored concomitant mutations from multiple pathways in above HER2-related signaling network (HER2 bypass signaling pathways, PI3K/Akt/mTOR pathway and TP53) were observed with significantly poorer PFS and OS when compared to those with none or one genetic alteration (median PFS, 7.3 vs. 26.1 months, *P* = 0.003; mean OS, 31.6 vs 56.1 months, median OS, 25.1 vs. 48.0 months, *P* = 0.013, Fig. 3B). However, no single alteration was correlated with significant OS difference except for ERBB2 mutation (8 patients, 19.0%, median OS, 15.5 vs 47.7 months, *P* = 0.001). Regarding PFS, significantly shorter PFS was observed in the patients with PIK3CA mutation (median PFS, 8.9 vs. 22.1 months, *P* = 0.013) and ERBB2 mutation (median PFS, 4.8 vs. 16.4 months, *P* = 0.010) when compared to those of wide-type (Supplementary Fig. 1).

## Discussion

In the past two decades, several small-molecule TKIs have demonstrated efficacy in HER2-positive metastatic breast cancer. Irreversible, pan-HER TKI contains neratinib and pyrotinib, are demonstrated with more complete inhibition of HER-family and promising antitumor activity in HER2-positive metastatic breast cancer patients compared to reversible TKI lapatinib. However, current data on the survival of irreversible TKIs was still limited and no previous study reported the survival outcome for different dose of irreversible TKIs or TKIs combined with chemotherapy in HER2-positive metastatic breast cancer. To our knowledge, this pooled analysis based on the updated individual survival outcome from phase I trials for pyrotinib and pyrotinib plus capecitabine is the first study to report the OS data on different dose of irreversible TKIs or TKIs combined with chemotherapy. Biomarker analysis via ctDNA in the baseline suggested concomitant mutations from multiple pathways in HER2-related signaling network may be a potential efficacy and prognosis biomarker for pyrotinib-base regimens in HER2-positive metastatic breast cancer.

Based on the updated survival data in the phase I trials, the median PFS was 8.2 months (95% CI: 5.6–10.9 months) in the pyrotinib monotherapy cohort and 22.1 months (95% CI: 14.3–29.9 months) in the pyrotinib plus capecitabine cohort, indicating promising antitumor activity in HER2-positive breast cancer. In the phase I trial for neratinib in solid tumor [12], the median PFS was 3.6 months (95% CI: 1.7 to 5.6 months) in patients with breast cancer. In the phase I/II trial of neratinib plus capecitabine in HER2-positive metastatic breast cancer [13], median PFS was 40.3 weeks (95% CI, 30.3 to 66.0 weeks) for patients with no prior lapatinib and 35.9 weeks (95% CI, 18.9 to 60.1 weeks) for those who had received prior lapatinib. Regarding the updated survival outcome in this study, the median OS was 27.1 months (95% CI: 21.6–32.5 months) in the pyrotinib monotherapy cohort and 37.4 months (95% CI: 12.2–62.7 months) in the pyrotinib plus capecitabine cohort. No previous phase I study of other TKIs in HER2-positive metastatic breast cancer reported on the outcome of OS. For neratinib, in the phase II trial [14] to compare neratinib monotherapy versus lapatinib plus capecitabine in HER2-positive metastatic breast cancer previously treated with trastuzumab and taxane, the median OS was 19.7 months in neratinib monotherapy group and 23.6 months in lapatinib plus capecitabine group (HR = 1.25;95% CI: 0.83–1.86; *P* = 0.280). Regarding neratinib plus capecitabine, the phase III NALA trial [8] in HER2-positive metastatic breast cancer previously treated with ≥2 HER2-directed regimens showed that no statistically significant difference was reached in OS between neratinib plus capecitabine group and lapatinib plus capecitabine (24.0 months vs 22.2 months; HR = 0.88; 95% CI:0.72 to 1.07; *P* = 0.2086). In the HER2CLIMB trial [15] for heavily pretreated patients with HER2-positive metastatic breast cancer, including those with brain metastases, the median OS was 21.9 months in the tucatinib plus trastuzumab combined with capecitabine and 17.4 months in the placebo plus trastuzumab combined with capecitabine (HR: 0.66; 95% CI: 0.50–0.88, *P* = 0.005). However, cross-trial comparisons could not be made directly due to the small sample size in this study and different enrolled criteria between these clinical trials.

Multiple mechanisms of primary or acquired resistance to HER2-directed therapies are reported to be associated with several signal transduction molecules in breast cancer, including dysregulation of downstream PI3K/Akt/mTOR signaling, PTEN loss, TP53 mutations, abnormal activation of other HER2 coligands (HER3, EGFR, IGFR), additional upregulation of HER2 via acquired amplifications and/or somatic mutations, etc. [16–20]. Due to significant molecular heterogeneity in HER2-positive breast cancer, a single biomarker may fail in capturing accurate picture of the heterogeneous cancer genome and helping to tailor therapy [16]. Currently, HER2 status is the only established predictive biomarker, regardless of extensive efforts in exploring biomarkers for the response to HER2-directed therapy [16]. In the biomarker analysis for pyrotinib monotherapy [7] and pyrotinib plus capecitabine [8], preliminary results indicate comprehensive analysis of concomitant mutations via ctDNA may be a potential biomarker for the therapeutic benefit of pyrotinib-based regimen. On basis of the updated survival data, this study further verified the patients harbored concomitant mutations from multiple pathways in HER2-related signaling network were associated with significantly worse progression-free survival and long-term survival when compared to those with none or one genetic alteration.

Though seldom previous research explored the clinical implications of concomitant mutations from multiple pathways in HER2-related signaling network for breast cancer, several previous studies [21–25] in EGFR-mutant advanced non-small cell lung cancer investigated the relationship between concomitant genetic alterations and survival outcomes. The results based on updated data in this analysis is consistent with previous studies [21–25], suggesting that significant concomitant genetic mutations may play a key role in drug resistance and tumor progression, and may be a significant factor affecting clinical survival outcomes [24]. Besides that, significantly shorter PFS was observed in the patients with PIK3CA mutation and ERBB2 mutation via the biomarker analysis of ctDNA when compared to those of wide type in this study. In the biomarker analysis based on primary or metastatic samples in phase III NALA trial, PIK3CA mutations trended toward shorter PFS, while ERBB2 mutations trended toward longer PFS. Previous studies showed biomarker analysis based on tumor samples may not neglect the effect of intratumor heterogeneity between metastatic specimens and primary tumors [26, 27]. However, the limited sample size in this biomarker analysis may not draw a confirm conclusion. Further investigation would be warranted to confirm the impact of concomitant genetic mutations from multiple pathways via ctDNA in a larger sample size from the following phase II and phase III trials. This pooled analysis included patients receiving pyrotinib monotherapy or pyrotinib plus capecitabine, and further research on the correlation between capecitabine and ctDNA mutations is warranted.

This study had several limitations. One of the main limitations of our study is related to the sample sizes. This analysis was based on the summary of individual patient data from two phase I trials, in which the sample size was limited to draw a confirm conclusion. Besides that, none of the patients have had pertuzumab or TDM1 before enrollment and only 65.2% of the patients (43 patients) had received prior trastuzumab treatment because during the recruitment periods of the two phase I clinical trials of pyrotinib (between February 2013 and November, 2015), pertuzumab was not approved in China, and access to trastuzumab was still relatively low. Additionally, these two phase I trials of dose escalation excluded the patients with brain metastases, and further study in HER2-positive breast cancer with brain metastases is warranted. However, to our knowledge, this description of survival outcome for pyrotinib or pyrotinib plus capecitabine in the phase I trials was the first attempt to depict the survival data for different dose of TKIs or TKIs combined with chemotherapy in HER2-positive metastatic breast cancer, which may provide a reference for those who were not tolerable to the recommended dose in clinical practice. Further final survival results from the phase II trial and the PHOEBE phase III trial would bring us more evidence on the survival benefit for irreversible pan-ErbB TKI.

In conclusions, this analysis of phase I trials for pyrotinib-based regimen from individual patient data revealed promising PFS and OS was achieved in patients with HER2-positive metastatic breast cancer. Concomitant mutations from multiple pathways in HER2-related signaling network via ctDNA may be a potential efficacy and prognosis biomarker for pyrotinib in HER2-positive metastatic breast cancer.

## Supplementary Information

**Additional file 1: Supplementary Fig. 1.** Kaplan-Meier estimates of (A) progression-free survival and (B) overall survival comparing patients ERBB2 mutation to those of wide-type. Kaplan-Meier estimates of (C) progression-free survival and (D) overall survival comparing patients PIK3CA mutation to those of wide-type.

## Data Availability

The datasets analyzed during the current study are available from the corresponding author on reasonable request.

## Abbreviations

HER2: Human epidermal growth factor receptor 2
MBC: Metastatic breast cancer
TKI: Tyrosine kinase inhibitor
EGFR: Epidermal growth factor receptor
PFS: Progression-free survival
OS: Overall survival
ATP: Adenosine triphosphate
ORR: Overall response rate
QoL: Quality of life
ctDNA: Circulating tumor DNA
