# Supplementary figures and images for: Survival benefit and biomarker analysis of pyrotinib or pyrotinib plus capecitabine for patients with HER2-positive metastatic breast cancer: a pooled analysis of two phase I studies
Source: Biomark Res. 2023 Feb 20;11:21. doi: 10.1186/s40364-023-00453-0 (PMC9940415; doi:10.1186/s40364-023-00453-0)

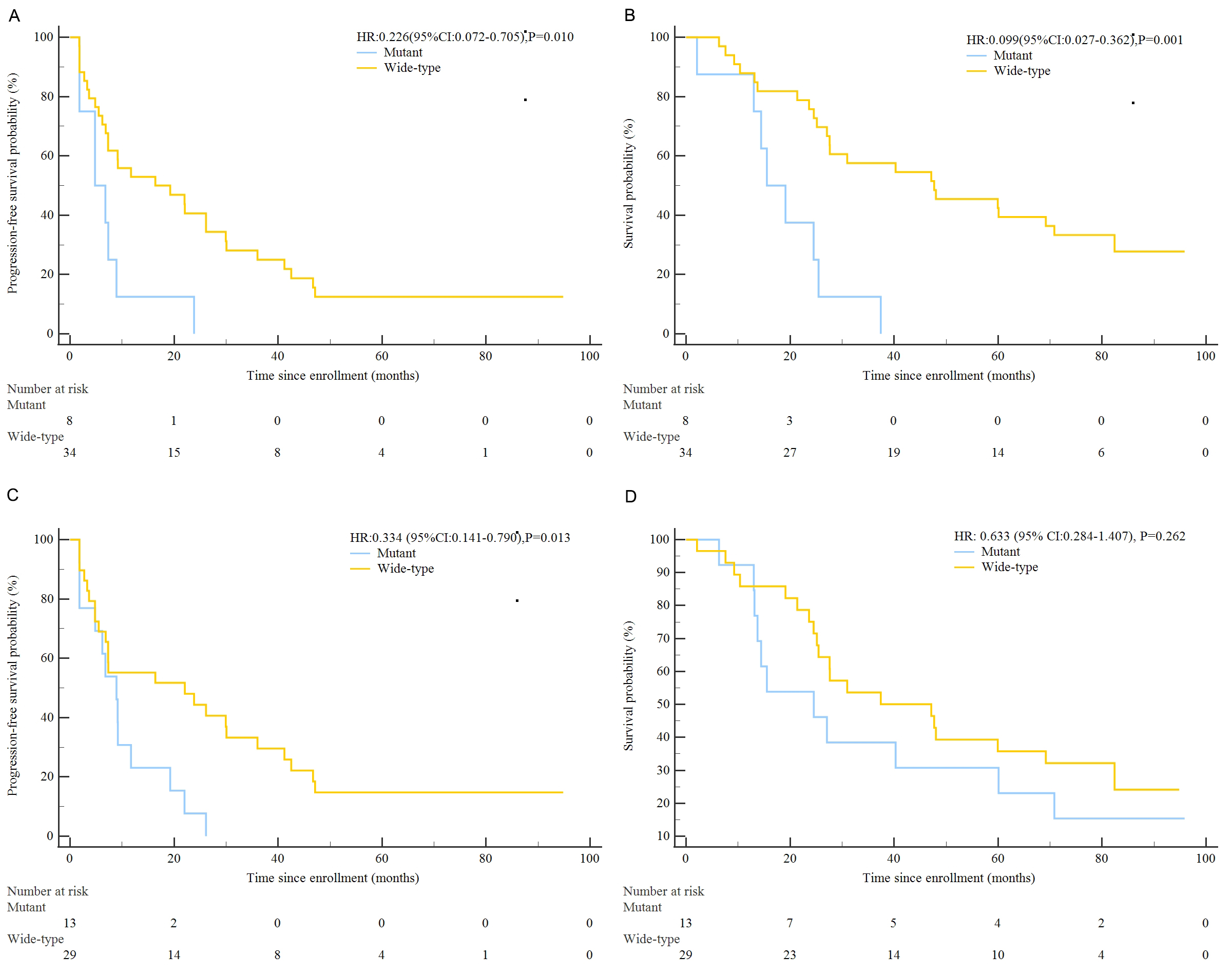

Supplement: Supplementary file 1 — Additional file 1: Supplementary Fig. 1. Kaplan-Meier estimates of (A) progression-free survival and (B) overall survival comparing patients ERBB2 mutation to those of wide-type. Kaplan-Meier estimates of (C) progression-free survival and (D) overall survival comparing patients PIK3CA mutation to those of wide-type. [file 40364_2023_453_MOESM1_ESM.jpg]
